# Long-term osteogenic differentiation of human bone marrow stromal cells in simulated microgravity: novel proteins sighted

**DOI:** 10.1007/s00018-022-04553-2

**Published:** 2022-10-01

**Authors:** Giulia Montagna, Giuseppe Pani, Dani Flinkman, Francesco Cristofaro, Barbara Pascucci, Luca Massimino, Luigi Antonio Lamparelli, Lorenzo Fassina, Peter James, Eleanor Coffey, Giuseppina Rea, Livia Visai, Angela Maria Rizzo

**Affiliations:** 1 Biochemistry Unit, Department of Molecular Medicine (DMM), Centre for Health Technologies (CHT), UdR INSTM University of Pavia, Pavia, Italy; 2 Department of Pharmacological and Biomolecular Sciences, Università degli Studi di Milano, via D. Trentacoste 2, 20134 Milan,, Italy; 3 Turku Bioscience Centre, University of Turku and Åbo Akademi University, Turku, Finland; 4 Department of Immunotechnology, Lund University Medicon, Medicon Village, Scheelevägen 2, 221 00 Lund, Sweden; 5 Institute of Crystallography CNR, via Salaria Km 29.300, Monterotondo, 00015 Rome, Italy; 6 Department of Gastroenterology, San Raffaele Hospital, via Olgettina 58, 20100 Milan, Italy; 7 Department of Electrical, Computer and Biomedical Engineering, Centre for Health Technologies (CHT), University of Pavia, via Ferrata 1, 27100 Pavia, Italy; 8 Medicina Clinica Specialistica, UOR5 Laboratorio di Nanotecnologie, ICS Maugeri, IRCCS, via Boezio 28, 27100 Pavia, Italy

**Keywords:** Simulated microgravity, Human primary cells, Osteogenic biomarkers, Cytoskeleton, Bone extracellular matrix, Bioimaging, Secondary osteoporosis, Proteomics, Data-independent acquisition

## Abstract

Microgravity-induced bone loss is a major concern for space travelers. Ground-based microgravity simulators are crucial to study the effect of microgravity exposure on biological systems and to address the limitations posed by restricted access to real space. In this work, for the first time, we adopt a multidisciplinary approach to characterize the morphological, biochemical, and molecular changes underlying the response of human bone marrow stromal cells to long-term simulated microgravity exposure during osteogenic differentiation. Our results show that osteogenic differentiation is reduced while energy metabolism is promoted. We found novel proteins were dysregulated under simulated microgravity, including CSC1-like protein, involved in the mechanotransduction of pressure signals, and PTPN11, SLC44A1 and MME which are involved in osteoblast differentiation pathways and which may become the focus of future translational projects. The investigation of cell proteome highlighted how simulated microgravity affects a relatively low number of proteins compared to time and/or osteogenic factors and has allowed us to reconstruct a hypothetical pipeline for cell response to simulated microgravity. Further investigation focused on the application of nanomaterials may help to increase understanding of how to treat or minimize the effects of microgravity.

**Supplementary Information:**

The online version contains supplementary material available at 10.1007/s00018-022-04553-2.

## Introduction

Since the 1970s space travelers have been known to experience severe bone loss at a rate of 1–1.5% per month, which is only partially responsive to non-pharmacological countermeasures [1, 2]. Pharmacological treatments, such as anti-resorptive bisphosphonates, do reduce bone loss in-flight but may interfere with the slow and often incomplete post-flight recovery process [3]. Thus, the exposure to a reduced gravity vector (or microgravity) during spaceflight is a significant and unresolved health risk for space travelers [4].

The bone is a metabolically active tissue in which the mineralized matrix is constantly deposited and resorbed by specialized cells. The extracellular matrix (ECM) is constituted by an organic portion, the osteoid, and an inorganic portion formed by different calcium phosphates, among which hydroxyapatite is the most represented. Bone marrow stromal cells (BMSCs) can differentiate into osteoblasts (OBs), which are cells specialized in the synthesis and deposition of the osteoid that is subsequently mineralized [5]. Osteoclasts (OCs) are, instead, devoted to the resorption of the mineralized matrix. The homeostasis between these cell types ensures a proper remodeling and turnover of the bone tissue. This is controlled by endocrine and paracrine factors, such as hormones and cytokines, which in turn are influenced by physiological, mechanical, and behavioral conditions [6–8]. Osteocytes (Ocys) residing within the lacunae of the mineralized bone are ultimately differentiated OBs secreting some of these factors, in function of the mechanical solicitation the whole tissue receives. Ocys are often referred to as the mechanosensors of the bone [9, 10].

Previous studies showed that exposure to real microgravity can impair OBs differentiation and mitochondrial energy potential, suppressing bone formation [11], and can abnormally increase the maturation and activity of OCs, prompting bone resorption [12, 13]. Thus, bone resorption-formation balance is disrupted during real microgravity exposure. However, molecular background knowledge of these mechanisms has not been completely understood yet.

Despite developments in space technology, the study of biological specimens in real space conditions is still a constraint. In this regard, ground-based platforms for simulating microgravity on Earth are an effective and invaluable asset. Among others, the random positioning machine (RPM) simulates weightlessness randomly, in a three-dimensional pattern, and represents a bench-top method for gravitational biology and space medicine research [14].

Previous reports have shown that ground-based simulated microgravity (SMG) reduces the osteogenic differentiation potential of BMSCs, although it is acknowledged that many of these studies were performed over short time frames, monitoring the effects of SMG for a maximum of 14 days [11, 15, 16]. Several studies have reported that SMG could enhance the multi-differentiation potential, namely the potential to differentiate into other cell types, of BMSCs [17, 18]. It has also been demonstrated that BMSC morphology is affected, changing from spindle to round shaped under SMG conditions, as a result of cytoskeletal rearrangement and which causes motility loss. Interestingly, Xue et al. found that the effect of SMG on the differentiation of BMSCs is dependent on exposure timespan. Short SMG exposure (72 h) enhanced the differentiation of BMSCs toward endothelial, adipogenic and neuronal lineages, while the longer period (10 days) could surprisingly promote the osteogenic differentiation of BMSCs [19]. These studies demonstrate that BMSCs, grown in vitro, at 72 h show a reduced adhesion capacity that is transduced with an increase in plasticity and multi-differentiation potential. However, at later time points, they acquire a more differentiated phenotype, according to the additional stimuli they receive. This is only partially in line with what is registered in vivo, in space travelers [20–22]. In their systematic review, Stavnichuk and coworkers highlighted that biochemical markers of bone resorption increased robustly to 113% above pre-flight levels, while bone formation markers increased slowly, at a rate of 6% per month, during spaceflight [4, 20–22], suggesting the influence of in vivo factors was modifying the BMSC response to microgravity. The in vivo situation was also different in terms of radiation exposure, as astronauts were sent into real space.

The present work aims to provide, for the first time, a comprehensive overview of the morphological, biochemical and molecular changes underlying the response of human BMSCs to long-term microgravity exposure (28 days) during osteogenic differentiation. In addition to expression profiling, analysis probing biomarker genes, bone matrix and cytoskeleton proteins, a study on bone mineralization was conducted along with a bottom-up, label-free, high coverage proteome investigation. Overall, our data pointed to an initial down-regulation of the osteogenic differentiation processes, highlighting proteins there were not identified before in SMG, and indicates that this down-regulation is partially reverted at later time points. This work provides research-based data for developing possible countermeasures against spaceflight-induced osteoporosis.

## Results

### Differentiation and mineralization of BMSCs in SMG

**Fig. 1:**
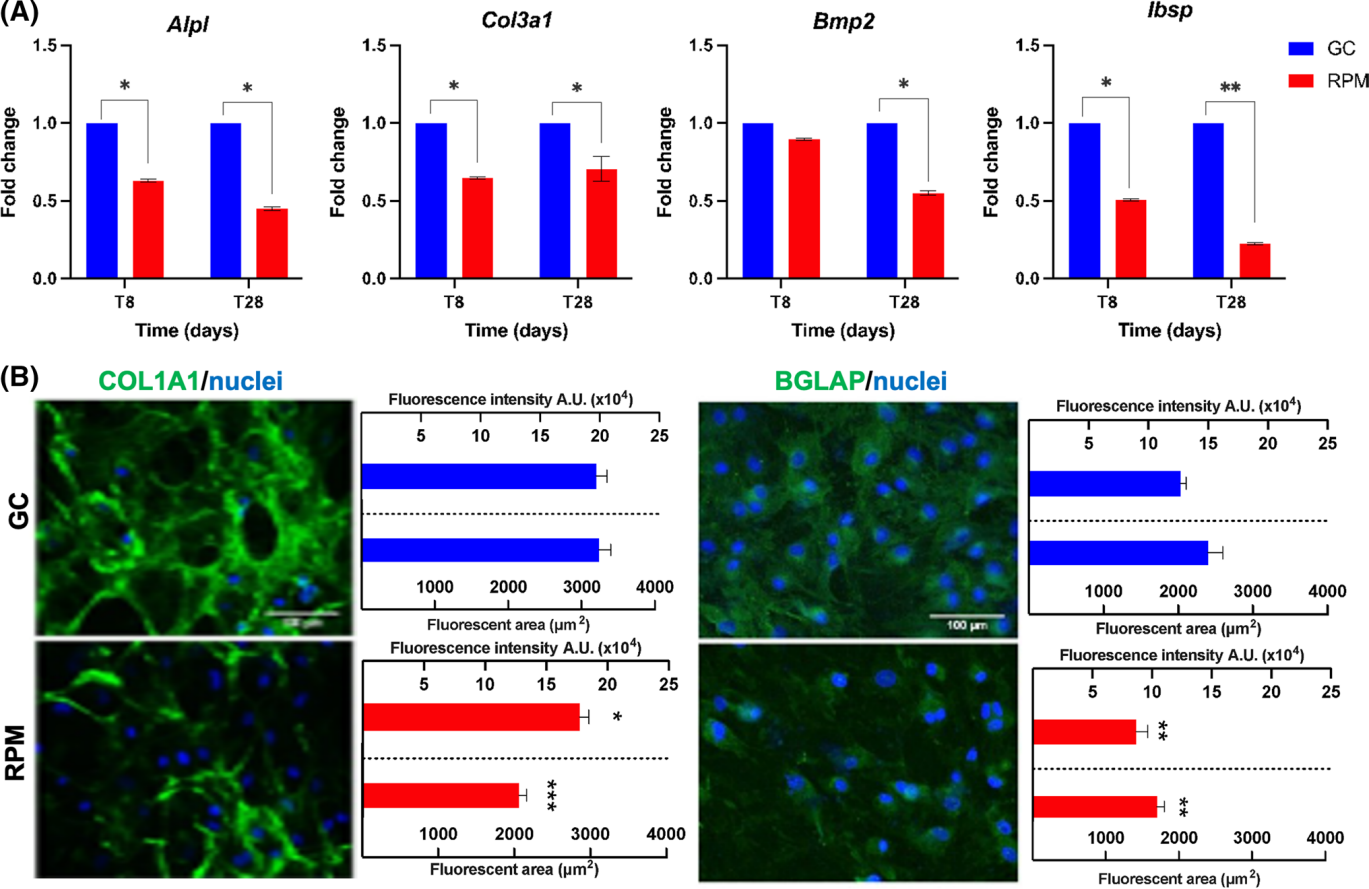
Osteogenic differentiation analysis by gene expression and extracellular matrix protein immunostaining. **A** Blue bars represent gene expression of Alpl, Col3a1, Bmp2 and Ibsp in GC condition, while red bars refer to RPM condition. Fold changes were calculated from the threshold cycles and expressed as the mean ± standard deviation. Student’s *T* test was applied for comparison against the gravity control GC. **B** Immunostaining at 28 days of differentiation showing collagen type I (COL1A1) marked in green with nuclei marked in blue on the left side of the panel and osteocalcin (BGLAP) marked in green with nuclei marked in blue on the right side of the panel, both in GC and RPM conditions. Blue bars represent the total fluorescence intensity (upper bars) and fluorescent area (lower bars) of the corresponding protein in GC. Red bars show the same parameters in the RPM condition. Statistically significant differences were assessed via Student *T* test (*T* tests: * = *p* < 0.05; ** = *p* < 0.01; *** = *p* < 0.001)

Exposure to SMG does not affect BMSC viability, either at 8 (T8) or 28 days (T28) (Fig. S1). The expression of osteogenic gene markers was assessed by qRT-PCR. Runt-related transcription factor 2 (*RUNX2*), alkaline phosphatase tissue non-specific (*ALPL*), decorin (*DCN*), and collagen type I (*COL1A1*) were analyzed as early markers, and collagen type III (*COL3A1*), bone morphogenetic protein 2 (*BMP2*), and bone sialoprotein 2 (*IBSP*), used as late markers. *RUNX2*, *DCN* and *COL1A1* were not significantly affected by RPM exposure (data not shown), while *COL3A1*, *ALPL*, *IBSP* and *BMP2* expression was downregulated by microgravity exposure at both T8 and T28 (Fig. 1A).

Differentiation was also evaluated at the protein level, by immunostaining of COL1A1 and the osteocalcin (BGLAP, also known as bone gamma carboxyglutamate protein) at 28 days (Fig. 1B) and by immunoenzymatic quantification of the bone ECM protein deposition at both time points (T8 and T28) (Table 1). Immunofluorescence analysis of both proteins, COL1A1 (Fig. 1B, left side) and BGLAP (Fig. 1B, right side), showed a significant reduction of fluorescence intensity and protein area in samples exposed to RPM compared to ground control (GC).

**Table 1:** Extracellular matrix protein evaluation by indirect ELISA

Proteins (μg/μg total proteins)	T8			T28			GC	RPM
	GC	RPM	GC vs RPM (*p*)	GC	RPM	GC vs RPM (*p*)	T8 vs T28 (*p*)	T8 vs T28 (*p*)
COL1A1	0.089 ± 0.004	0.091 ± 0.002	0.563	0.189 ± 0.019	0.123 ± 0.008	*0.045	*0.018	*0.030
COL3A1	0.390 ± 0.016	0.387 ± 0.013	0.850	0.409 ± 0.001	0.411 ± 0.006	0.684	0.243	0.142
DCN	0.261 ± 0.030	0.257 ± 0.010	0.879	0.258 ± 0.006	0.257 ± 0.021	0.961	0.898	0.994
FN	12.65 ± 0.213	13.12 ± 0.414	0.296	31.47 ± 27.76	20.82 ± 21.80	0.711	0.439	0.667
BGLAP	20.52 ± 2.095	23.58 ± 0.178	0.176	19.91 ± 0.943	20.10 ± 1.435	0.893	0.744	0.077
SPARC (× 10^3^)	8.308 ± 0.184	8.761 ± 1.095	0.623	8.449 ± 0.567	9.144 ± 0.223	0.248	0.771	0.675
SPP1 (× 10^3^)	114.4 ± 3.121	132.0 ± 1.112	*0.017	118.2 ± 0.646	118.6 ± 3.013	0.869	0.231	*0.027

In bold are evidenced the values showing significance

Indirect enzyme-linked immunosorbent assay (ELISA) quantifying the amount of specific protein detected per μg of total protein content. Statistical significance was assessed via Student *T* test, comparing the GC and RPM conditions at both time points. In the column *p*, the *p* value is reported. * = *p* < 0.05

Immunoenzymatic quantification was done on COL1A1, COL3A1, DCN, fibronectin (FN), BGLAP, osteonectin (SPARC), and osteopontin (SPP1, also known as secreted phosphoprotein 1) (Table 1). The only statistically significant variations were seen in the levels of COL1A1 and SPP1. In detail, COL1A1 was found upregulated at 28 days with respect to 8 days, both in GC and RPM conditions (Table 1, GC—T8 vs T28 and RPM—T8 vs T28), indicating osteogenic differentiation was undergoing in both conditions. Although, at 28 days in RPM, the COL1A1 content was lower than in GC condition (Table 1, T28—GC vs RPM), suggesting a reduced osteogenic differentiation. SPP1 was found upregulated at 8 days in RPM with respect to 8 days in GC condition (Table 1, T8—GC vs RPM). However, the SPP1 content at 28 days in RPM was lower than at 8 days in RPM (Table 1, RPM—T8 vs T28). The immunoenzymatic quantification of COL3A1, DCN, FN, BGLAP and SPARC showed non-significant variations among the classes. Taken together these results indicate lower or delayed differentiation of BMSCs when grown on the RPM.

**Fig. 2:**
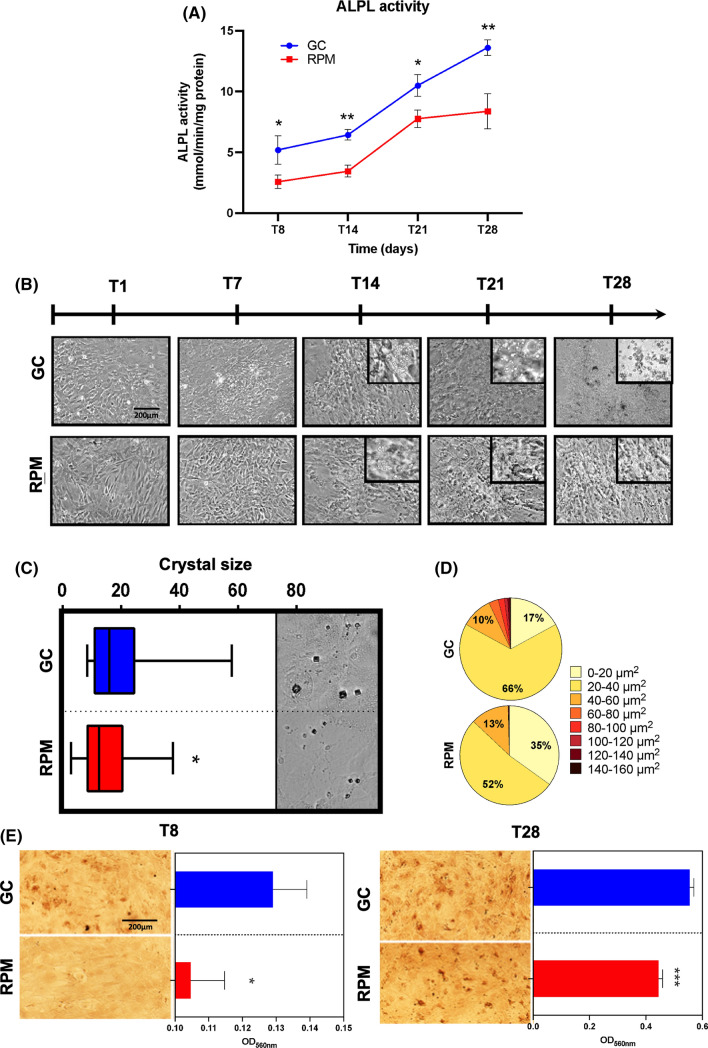
Mineralization assessment of BMSCs during osteogenic differentiation in GC and RPM. **A** ALPL enzymatic activity assessment at 4 time points: 8, 14, 21 and 28 days, in GC and RPM. **B ** Optical microscope visualization of differentiating cells throughout the 28 days of experiment and crystal formation from the 14th day (inserts). **C** Maximum, minimum, and average crystal size values at 28 days are reported in the box plot and supplied with qualitative figures of the corresponding crystals. Statistics was calculated on more than 60 crystals per experimental point in images acquired with 20 × magnification in phase contrast via Student *T* test, comparing crystals in GC to RPM. **D** Frequency distribution of crystal size in GC and RPM conditions at 28 days. **E** Qualitative and quantitative representation of the alizarin red staining performed at 8 (T8) and 28 days (T28) in GC (blue histograms) and RPM conditions (red histograms). Significant differences were assessed via Student *T* test (*T* tests: * = *p* < 0.05; *** = *p* < 0.001)

ALPL enzymatic activity was significantly lower in SMG at four investigated time points over the 28 days (Fig. 2A) in agreement with ALPL transcript expression, which is also downregulated in RPM at T8 and at T28 (Fig. 1A). Additionally, the mineralization of the ECM determined by measuring crystal size revealed a significant reduction in the dimension and an altered percentage distribution (Fig. 2B–D), suggesting impaired osteoblasts activity. Indeed, on RPM, it was already possible to observe smaller crystals at T14 compared to GC conditions. Alizarin red positive spots were almost completely absent in RPM at T8 but they were present at T28, again indicating impaired osteoblasts activity. In accordance, alizarin red absorbance showed a significant reduction of calcium deposits at T8 and T28 in RPM samples (Fig. 2E). These results indicated a reduction in the osteogenic phenotype when BMSCs were exposed to SMG. However, the synthesis of some ECM proteins and the mineralization capacity of the differentiating BMSCs were not completely impaired.

### Cytoskeleton investigation with bio-imaging in SMG

**Fig. 3:**
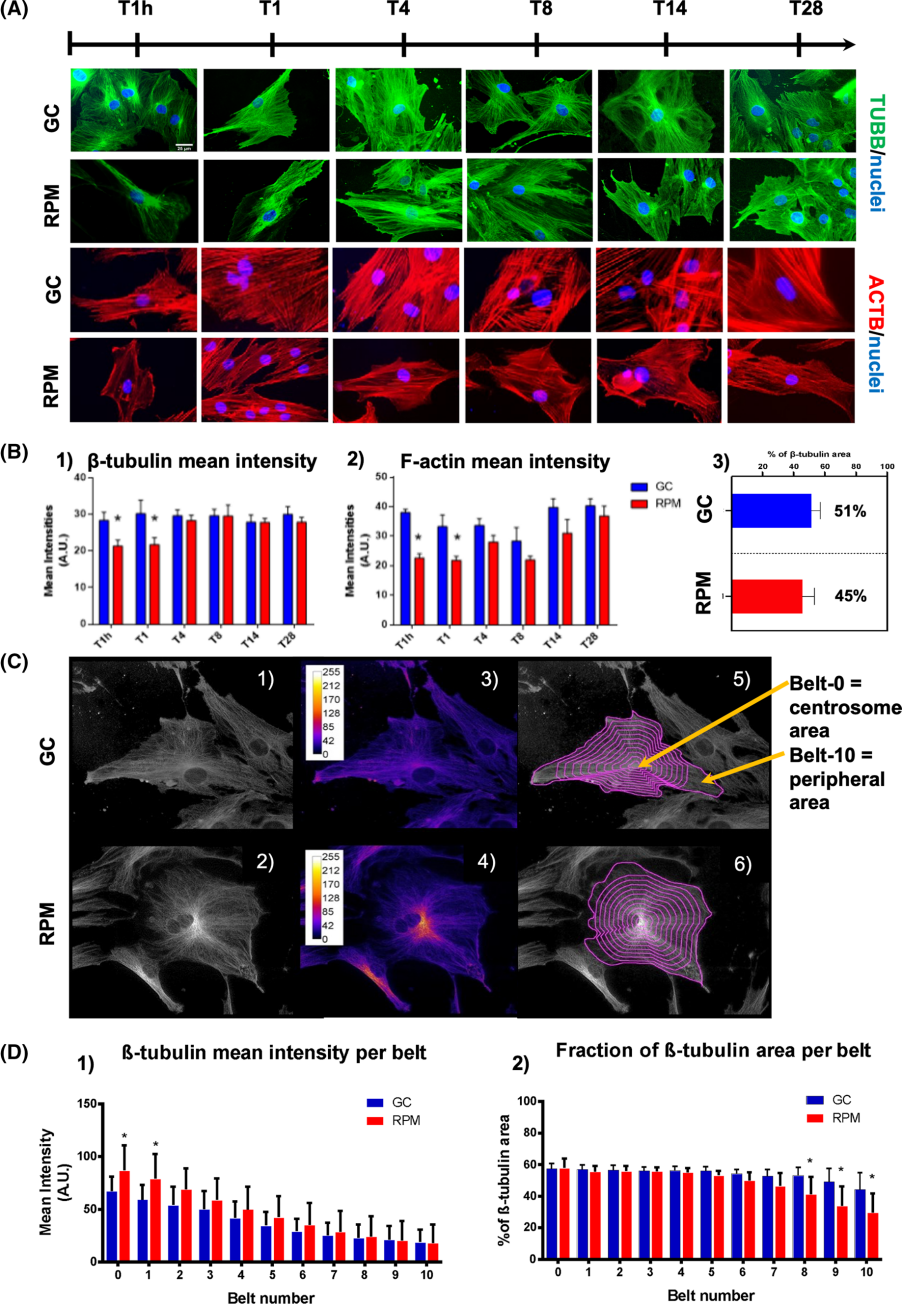
Cytoskeletal reorganization investigated by immunostaining and bio-imaging analysis. **A** β-tubulin (green), F-actin (red) and nuclei (blue) were stained and visualized following 1 h, 1, 4, 8, 14, and 28 days of differentiation, both in GC and RPM conditions. **B** Graphs of F-Actin and β-tubulin mean intensity (*T* tests: * = *p* < 0.05)**. C** Gray-scale images (1 and 2), fire lookup table (3 and 4) and concentric belts into cells (5 and 6) were used for the overall distribution of tubulin fluorescence intensity. The concentric distribution algorithm masks were used to quantify microtubules distribution per belt (5 and 6) and 15 cells per field were analyzed in three experimental replicates. **D** Graph of mean intensity and percentage of β-tubulin into each belt (Belt 0 = close to the centrosome; belt 10 = cell periphery) (*T* tests: * = *p* < 0.05)

**Fig. 4:**
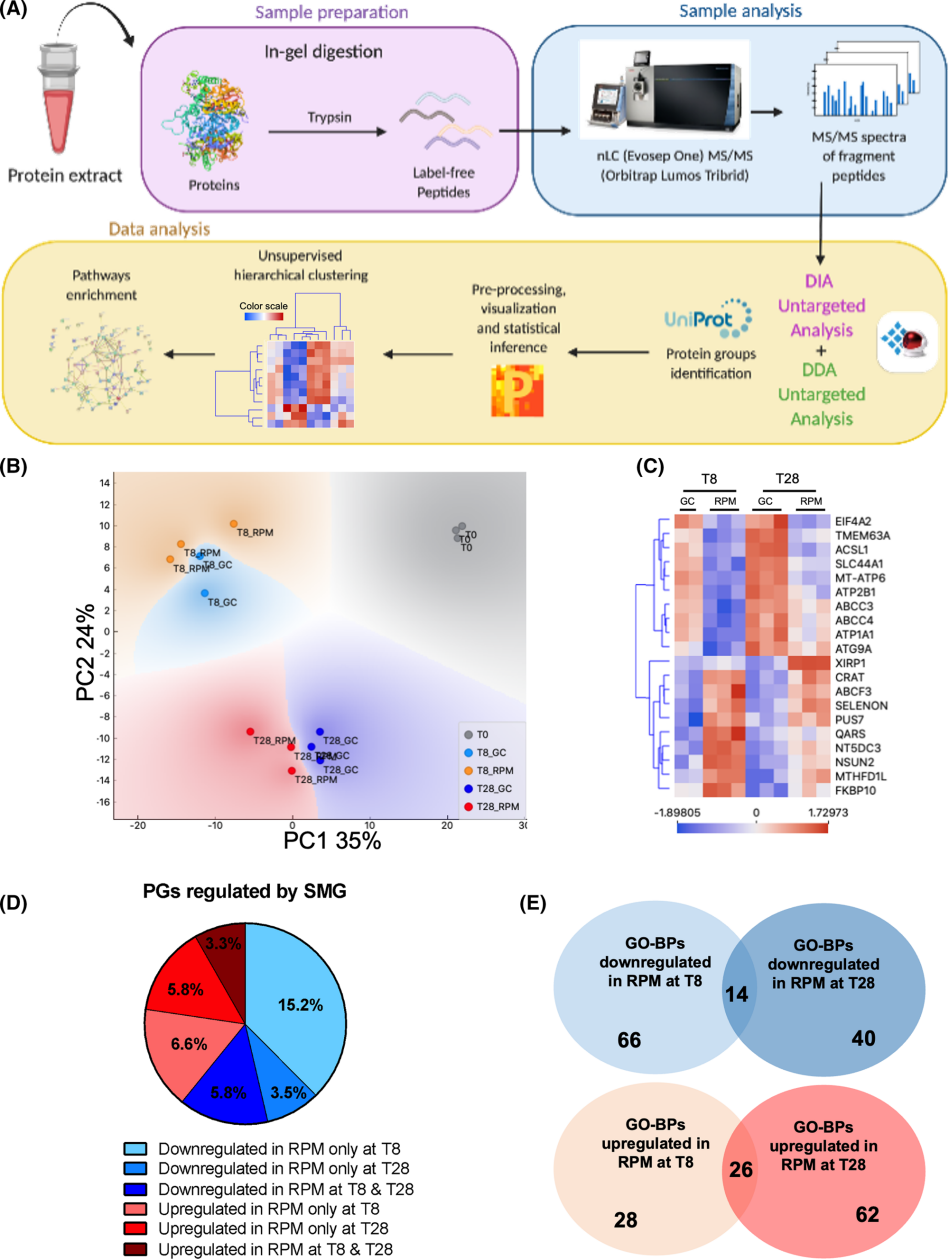
Proteomics investigation. **A** Schematic representation of the experimental proteomics workflow, with indications of the software utilized in the different steps. **B** PCA of the sample replicates based on the protein groups intensities characterizing each sample. PC1 explains 35% of the variance and PC2 24%. **C** Hierarchical clustering of the top ten most downregulated and ten upregulated PGs **D** Pie chart showing DAPGs divided by their trend in RPM with respect to GC condition. **E** Following enrichment analysis of the DAPGs, a total of 90 pathways were found upregulated and 106 downregulated

**Fig. 5:**
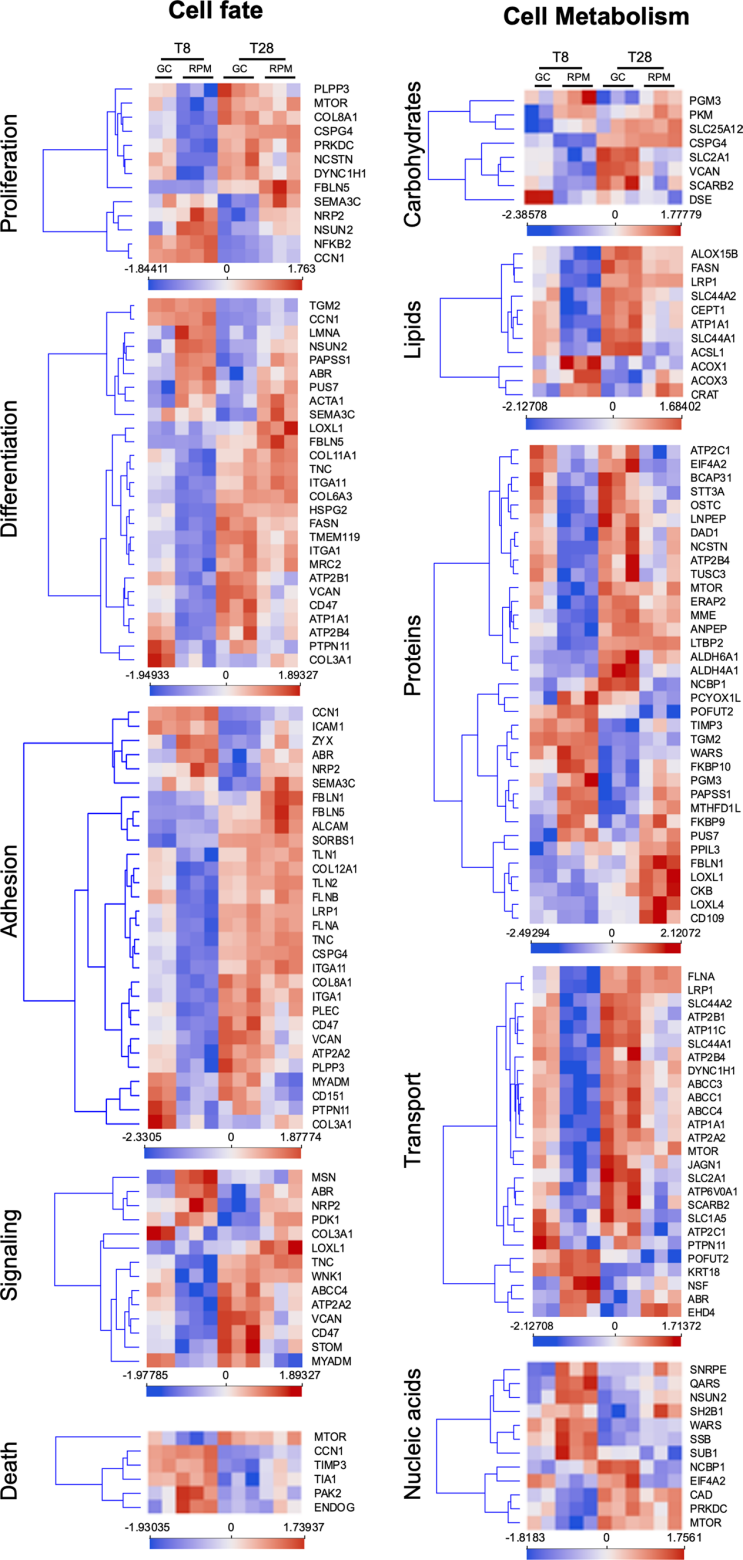
Differentially abundant protein groups (DAGPs) categorization under major GO biological processes. The enriched GO_BPs were categorized under 10 major classes mainly involved in cell metabolism and cell fate. Data mapped are the Z-scored of the ratioed intensities

F-Actin and β-tubulin were investigated on BMSCs during osteogenic differentiation in order to determine the long-term effect of SMG on cytoskeleton proteins by immunofluorescence (Fig. 3A). Intensity analysis of the cells revealed that during the first 24 h of microgravity, there was a significant decrease in both tubulin and actin mean intensity; actin filaments (Fig. 3B-1) and the tubulin network (Fig. 3B-2) showed a recovery after 4 days. Indeed, after 28 days, the percentage of tubulin area in cells exposed to SMG was not significantly different compared to GC (Fig. 3B-3). Deeper analysis, initially performed with “fire lookup table” (Fig. 3C-3 and C-4) and later with mean intensity and fraction area measurements per belt, revealed that distribution of β-tubulin within cells was still altered after 28 days. In particular, the mean intensity of samples exposed to SMG was significantly higher in proximity to the centrosome, and the surface occupied by tubulin was lower in the cell periphery (Fig. 3C-5, C-6, D-1 and D-2). This evidence shows that a cytoskeletal adjustment of β-tubulin and F-actin fibers occurred during the first two days of SMG exposure. However, these changes were almost completely reversed at 28 days, leaving the cells with a reduced β-tubulin fluorescent area in their more peripheral cytosolic portion.

### Proteomics investigation of the protein groups affected by SMG

To understand proteome variations caused by SMG during the osteogenic differentiation of BMSCs, we applied a proteomics approach based on mass spectrometry label-free quantification (Fig. 4A). The proteomics investigation allowed the identification of 4312 protein groups (PGs) in total (Table S1). Following data preprocessing (ratio on time zero, T0, filtering for valid values, log2 transformation, and missing data imputation), the number of PGs was reduced to 4114. To narrow down the number of PGs relevant for this study, multiple sample tests were used to statistically infer the differentially abundant PGs (DAPGs). To simplify the statistical model, and following data interpretation, the two independent variables present in the experiment (time and SMG exposure) were fused into one unique independent variable. The classes of the unique independent variable were therefore four: T8_GC (BMSCs in osteogenic differentiation for 8 days under gravity control), T8_RPM (BMSCs in osteogenic differentiation for 8 days under SMG), T28_GC (BMSCs in osteogenic differentiation for 28 days under gravity control), and T28_RPM (BMSCs in osteogenic differentiation for 28 days under SMG). 486 PGs, reduced to 481 following isoforms check, were differentially abundant among the four classes, and post hoc THSD was applied to annotate class-specific variation for each DAPG (Table S2).

Principal component analysis (PCA) of the sample replicates showed that 59% of variance was due to the first two components (Fig. 4B). The replicates were clustered by the PCA into five groups, based on the two independent variables (time and SMG exposure). T8 groups appeared more distant from T28 groups than GC from RPM, suggesting that time was generating more variance than SMG on BMSCs proteome.

GO annotation overrepresentation analysis (ORA) of the 481 DAPGs showed that 13 cellular component categories (GO-CC) were significantly enriched (Fig. S3). Interestingly, 49 DAPGs were mapped to the GO-CC ECM (*p* = 2.7 × 10^–11^), while 28 were mapped to the mitochondrial matrix (*p* = 3.1 × 10^–3^), 47 to the cell-substrate junction (*p* = 3.8 × 10^–13^), and 30 to the actin cytoskeleton (*p* = 1.5 × 10^–3^).

Z-scored values of the top 10 PGs most downregulated (ABCC3, ABCC4, ACSL1, ATG9A, ATP1A1, ATP2B1, EIF4A2, MT-ATP6, SLC44A1, TMEM63A) and the top 10 PGs most upregulated (ABCF3, CRAT, FKBP10, MTHFD1L, NSUN2, NT5DC3, PUS7, QARS, SELENON, XIRP1) in RPM were hierarchically clustered (Fig. 4C). Interestingly, many of these 20 DAPGs are under the control of stemness master regulators, such as POU5F1 and NANOG.

To understand how the SMG exposure was affecting PGs regulation over time, the 481 DAPGs were divided in a pie chart (Fig. 4D) based on their regulation in SMG at 8 and 28 days. Of those 481 DAPGs, 15.2% (73 PGs) and 6.6% (32 PGs) were respectively down- and upregulated exclusively at T8, against the 3.5% (17 PGs) and 5.8% (28 PGs) that were, respectively, down- and upregulated solely at T28. An additional 5.8% (28 PGs) and 3.3% (16 PGs) were found down- and upregulated at both time points. The remaining 59.8% of DAPGs were attributed to the BMSCs maturation during time, comparing the conditions T8_GC vs T28_GC and T8_RPM with T28_RPM. This distribution highlighted the fact that a reduction in the overall metabolism of the BMSCs underwent at early time points (8 days) and was relieved at the 28th day. An increasing upregulation was instead present in these cells.

The enrichment analysis resulted in overrepresentation of 178 unique GO-BP terms (Table S3) and helped to narrow down the cellular processes mainly affected by SMG. These 178 GO-BPs were represented in four charts based on their down- and upregulation data, at 8 and 28 days (Fig. S4–S7). They presented redundant DAPGs in their list, but this also ensured that the biological significance of the proteins was not underestimated (as one protein can be, and usually is, involved in multiple pathways).

In general, the overall proteomics coverage was high with respect to previous works (4312 identified PGs). However, the GO-CCs annotation of the DAPGs highlighted a polarized enrichment of non-nuclear proteins. Results from the 481 DAPGs suggest that major changes occurred at early time points (8 days), with the 15.2% of all DAPGs being downregulated by SMG at 8 days.

### Functional annotation of the DAPGs and relative enriched GO-BPs found in SMG

To rationalize the GO-BPs revealed by enrichment analysis, we clustered them into two macro-categories, each comprising five categories which were functionally correlated. The *cell fate* macro-category included proliferation, differentiation, adhesion, signaling, and death (Fig. 5). The *cell metabolism* macro-category, on the other hand, was divided in carbohydrates, lipids, proteins, nucleic acids metabolisms, and transport (Fig. 5). Proteomics data showed the enrichment of 13 GO-BPs related to BMSCs and osteoblasts *proliferation*. Among the enriching proteins, a significant reduction at 8 days in the expression of seven DAPGs (COL8A1, CSPG4, DYNC1H1, MTOR, NCSTN, PLPP3, and PRKDC) was followed by a significant upregulation at 28 days of 6 DAPGs (CCN1, FBLN5, NFKB2, NRP2, NSUN2, and SEMA3C) (Fig. 5—Proliferation). Notice that CCN1 and FBLN5 were annotated to the GO-BP “positive regulation of osteoblast proliferation” (GO:0033690). MTOR and PRKDC can also be associated with the PI3K/AKT/mTOR pathway.

16 out of 27 DAPGs under the *differentiation* category (Fig. 5—Differentiation) were downregulated in RPM at 8 days (ATP1A1, ATP2B1, ATP2B4, CD47, COL11A1, COL3A1, COL6A3, FASN, HSPG2, ITGA1, ITGA11, MRC2, PTPN11, TMEM119, TNC, and VCAN), and only 6 of these were downregulated at 28 days (ATP1A1, ATP2B1, CD47, FASN, TMEM119, VCAN). All of these are annotated under or related to the GO-BP terms “osteoblast differentiation, ossification, skeletal system morphogenesis and bone development.” The other 11 DAPGs were found upregulated in RPM: 6 were upregulated at the earliest time point (ABR, ACTA1, LMNA, NSUN2, PAPSS1, PUS7) and 10 at the last time point (ABR, ACTA1, CCN1, FBLN5, LOXL1, NSUN2, PAPSS1, PUS7, SEMA3C, TGM2). Interestingly, MAP2K1 (also known as MAPKK 1, MKK1, ERK activator kinase 1, MAPK/ERK kinase 1, or MEK1) was found downregulated at 28 days with respect to 8 days, both in RPM and GC (Table S2). MAP2K1 can inhibit PPARG activity in the nucleus and repress adipogenic differentiation. A non-significant increase in MAP1K2 in RPM with respect to GC might suggest a mild reduction in adipogenesis (Table S2).

In the *adhesion* category (Fig. 5—Adhesion), there were 30 enriched GO-BPs. Among them, 14 were downregulated in RPM at 8 days (ATP2A2, CD151, CD47, COL12A1, COL3A1, COL8A1, CSPG4, FLNA, FLNB, ITGA1, ITGA11, LRP1, MYADM, PLEC, PLPP3, PTPN11, TLN1, TLN2, TNC, VCAN) and 5 of these were also downregulated at 28 days (CD151, CD47, COL8A1, MYADM, VCAN), indicating a reduction in overall cell adhesion and migration properties. Interestingly, the downregulated DAPGs enriched for the integrin-mediated signaling pathway (GO:0007229 by CD47, ITGA1, ITGA11, PLPP3, and PTPN11), hemidesmosome assembly (GO:0031581 by CD151 and PLEC), cytoskeleton organization (GO:0007010 by FLNB, TLN1, and TLN2) and ECM organization (GO:0030198 by COL8A1, TCN, and VCAN), suggest a reduction in BMSC adhesion and an overall reorganization of the cytoskeletal fibers at the early time point (8 days). ABR, SORBS1, and ZYX were upregulated at 8 days in RPM and 7 were upregulated at 28 days (ALCAM, CCN1, FBLN1, FBLN5, ICAM1, NRP2, SEMA3C). The early (8 days) upregulation of SORBS1 and ZYX, enriching for stress fiber assembly, was in line with results derived from F-actin bioimaging analysis. Indeed, following a sudden reduction in stress fibers (shown as reduction in the mean fluorescent intensity) at T1h (Fig. 3A), there was a reorganization and reformation of those fibers during the following days (fluorescent area and intensity increased already at day 4 and remained comparable to GC condition) (Fig. 3A-B). The later upregulation of DAPGs involved in cell migration (ALCAM, NRP2, SEMA3C) suggests BMSCs started migrating again once they reorganized the cytoskeleton and adapted to SMG.

In the *signaling* category (Fig. 5—Signaling), a total of 14 DAPGs were found, of which 8 were downregulated at 8 days (ABCC4, ATP2A2, CD47, COL3A1, MYADM, TNC, VCAN, WNK1) and 5 remained downregulated at 28 days. While 2 were upregulated at 8 days (ABR and MSN) and 4 were upregulated at 28 days in RPM (ABR, LOXL1, NRP2, PDK1). Among the proteins enriching the signaling category, 8 were also found to enrich the adhesion category (Fig. 5—Adhesion) (ABR, ATP2A2, CD47, COL3A3, MYADM, NRP2, TNC, VCAN). This was possibly due to the fact that a reduction in cell adhesion and cytoskeletal reorganization events was the stimulus and/or response to the microgravity signal.

6 DAPGs enriched the *cell death* category (Fig. 5). Among them MTOR was downregulated at 8 days, ENDOG and PAK2 were upregulated at 8 days, and CCN1, TIA1 and TIMP3 were upregulated at 28 days, indicating an overall upregulation of apoptosis. This is in line with the data on cell viable numbers, as we registered an almost constant number of alive BMSCs, due to a certain percentage of proliferation and a certain percentage of apoptosis.

Regarding the *cell metabolism* macro-category (Fig. 5), we grouped the DAPGs involved in the metabolism of the different macromolecules. *Carbohydrate* metabolism (Fig. 5—Carbohydrate) included simple and complex carbohydrates metabolic processes. In this category, CSPG4, DSE and VCAN were downregulated at 8 days, while SCARB2, SLC2A1 and VCAN were downregulated at 28 days. These proteins mostly enriched the biosynthetic pathways of lactose, chondroitin and dermatan sulfate biosynthesis (the last two being glycosaminoglycans—GAGs). PKM and SLC25A12 were upregulated at 8 days and PGM3 only at 28 days, which enriched glycolysis-related GO-BPs. Interestingly, PKM and SLC2A1 were also involved in the aerobic glycolysis pathway. PDK1 is upregulated at 28 days in RPM with respect to GC, and PDK3 and PC are upregulated at 28 days with respect to 8 days, in both RPM and GC (Table S2). PFKL and PFKP were downregulated in both conditions at 28 days with respect to 8 days (Table S2).

Among the 11 DAPGs enriching *lipid-*related metabolic processes (Fig. 5—Lipids), 8 were downregulated at 8 days (ACSL1, ALOX15B, ATP1A1, CEPT1, FASN, LRP1, SLC44A1, SLC44A2) and 6 at 28 days (ACSL1, ALOX15B, ATP1A1, CEPT1, FASN, SLC44A1). These downregulated proteins generally enriched lipid or lipidated molecules biosynthesis-related pathways. Only ß-oxidation-related proteins were found upregulated (ACOX1 and CRAT at 8 days and ACOX3 and CRAT at 28 days), and the leptin receptor (LEPR) was found upregulated at T28 in RPM with respect to T8 in RPM (Table S2), suggesting an increase in the basal metabolism undergoing in RPM at 28 days. Furthermore, ACADM, which is the catalyst for the first step of mitochondrial fatty acid beta-oxidation, was upregulated at 28 days in both conditions, while ACAA2, the catalyst for the last step of the mitochondrial beta-oxidation pathway, was downregulated at 28 days with respect to 8 days in both conditions (Table S2). So, overall lipid-related proteins suggest a general downregulation of lipid biosynthesis and upregulation of lipid degradation by oxidation, which is in line with a general spike of the basal metabolism.

In the *protein metabolism*-related pathways (Fig. 5—Proteins), we found a total of 35 DAPGs. The DAPGs involved in protein N-glycosylation (DAD1, OSTC, STT3A, TUSC3), regulation of translational initiation (EIF4A2, MTOR, NCBP1) and other protein modification processes were downregulated in RPM, while DAPGs involved in the elastic fiber formation processes were upregulated (FBLN1, LOX1, LOX4, TIMP3). The synthesis of biglycan (BGN), bone-related proteoglycan, is significantly increased in RPM at 28 days (Table S2).

In the *transport*-related processes (Fig. 5—Transport), we annotated 26 different DAPGs, and 22 of these were downregulated against the four DAPGs upregulated by SMG (ABR, EHD4, KRT18, NSF). 10 of the downregulated DAPGs were annotated to pathways of export from the cells and 4 are involved in calcium ion transport (ATP1A1, ATP2B1, ATP2B4, and ATP2C1).

Regarding the *nucleic acids* metabolic processes (Fig. 5—Nucleic acids), we found several DAPGs involved in RNA modification upregulated (NSUN2, QARS, SNRPE, SSB, WARS) in RPM, while DNA metabolic processes were mostly linked to DAPGs downregulated by RPM (CAD, MTOR, PRKDC). In contrast to the general tendency of having more downregulated proteins in RPM, in this category composed of 12 DAPGs, only 5 were downregulated and 7 upregulated.

## Discussion

Characterizing the effects of microgravity exposure on in vivo models is challenging and is limited by a number of factors. The design and development of ground-based microgravity simulators, such as RPM, has improved the chances for microgravity study and allowed the in-house exposure and analysis of biological samples [14].

Contrasting data on osteogenic differentiation of BMSCs are present in the literature due to a remarkable variability attributed both to the experimental settings and the model used [4, 23].

In our study, proteomics data and cell viability suggested that apoptosis was occurring to some extent but that it was not significant, as previously indicated by Wang et al. [17]. Proliferative processes (also enriching the PI3K/AKT/mTOR pathway [24]) were reduced at 8 days, followed by an upregulation at 28 days of other DAPGs involved in proliferation and specifically osteoblast proliferation pathways, suggesting a “change of course” for BMSCs. This is partially in line with what was found by Bucaro et al. [23], even if their experimental model was different from ours: they used MC3T3-E1 osteoblast-like cells included in alginate carriers and NASA-engineered high aspect ratio vessel (HARV).

SELENON, a protective component against oxidative stress, was upregulated in the present study, suggesting the involvement of oxidative stress in SMG, as previously reported [11, 25, 26].

Chen et al., showed that rat BMSCs, analyzed on a 2D-clinostat for 48 h, showed F-actin depolarization and that this was reflected on the inactivation of TAZ and consequent TAZ impairment translocating to the nucleus. TAZ is a transcription factor involved in the osteoblastic differentiation of BMSCs; therefore, its inactivation affected BMSCs differentiation capacity [15, 27]. Similarly, in our study, we identified a reduced mean intensity of F-actin fibers, supporting the F-actin depolarization conclusion, and found that BMSCs exhibited a reduced, but not completely impaired, osteoblastic differentiation capacity. Cytoskeletal rearrangements were studied in a different work, in connection with mTOR regulation in murine BMSCs, and it was shown that mechanical stress elicited an increase in the mTOR synthesis, causing a stiffening of the cytoskeleton and thereby promoting osteoblastic differentiation [28]. Decreased expression of MTOR was caused by SMG and this might trigger the cytoskeletal rearrangement. Reduced gene expression markers and protein markers for osteoblastogenesis, both at early and late time points, indicated a general decline of this differentiation process, as shown by previous studies [29]. Furthermore, MAP2K1 is involved in the promotion of adipogenesis by exporting PPARG transcription factor from the nucleus to the cytoplasm and reducing its gene regulatory activity [30]. Its downregulation (MAP2K1) in time was present in both experimental conditions, and we found a non-significant increase in SMG, suggesting a mild reduction in the adipogenic differentiation. Indeed, genes controlled by PPARG, like FABP3, and PLIN2 [31] and involved in adipogenesis, such as AEBP1 [32], were not upregulated by SMG. Rather, their expression was promoted with time, in both GC and RPM conditions.

Proteomic data also showed a reduction in the enzymes involved in protein synthesis, glycosylation of proteins and synthesis of glycosaminoglycans (GAGs) (Fig. 5—Carbohydrates). GAGs play a pivotal role in connective tissues, including in the case of bone and cartilage [33]. They are crucial for the organization of the ECM and for the signaling from the ECM to the cells. DCN concentration measured by enzyme-linked immunosorbent assay (ELISA) did not register significant differences, although the dermatan sulfate epimerase (DSE) enzyme was found downregulated, suggesting an impaired glycosylation of DCN [34]. Novel markers of osteoblastic differentiation of human BMSCs recently identified by other studies include SLC44A1 (also known as CD10) [35] and MME (also known as CD92) [35]. Interestingly, these markers were found to increase during the osteogenic and adipogenic differentiation of BMSCs [35, 36], and our results showed that these proteins were downregulated under SMG (Fig. 4C and Fig. 5—Proteins, respectively). CD109 was also associated with mature OBs [37], and PTPN11 KO mice exhibited osteopenia phenotypes [38]. We reported their downregulation at 8 days and upregulation or non-significant variations at 28 days, in SMG with respect to GC.

ALPL is an early osteogenic differentiation marker [39] whose expression and biosynthesis is constantly upregulated in differentiating osteoblasts up to the 28th day of differentiation [40]. Its activity is essential for the mineralization of the ECM since it provides the inorganic phosphate (P_i_) for hydroxyapatite crystal growth [41]. The downregulation of its gene expression and activity under SMG is congruent with the reduction in matrix mineralization. Furthermore, *ALPL* expression under SMG is downregulated with respect to GC, but it is not completely erased. Interestingly, we found TMEM63A among the ten mostly downregulated in differentiating BMSCs under SMG, and there are very few citations linking TMEM63A to the bone in the current literature [42, 43]. TMEM63A is a mechanosensitive cation channel capable of converting the mechanical stimulus of pressure into a calcium flow [44]. Interestingly, Sun and colleagues showed a reduced intracellular free calcium concentration in mouse primary osteoblasts under simulated microgravity [45]. We can speculate that this mechanosensitive calcium channel might be linked to the mechanotransduction of the SMG, by reducing the calcium flow entering the cells. Consequently, when mineralization was assessed in terms of crystal number, crystal size and alizarin red staining, it was found reduced in SMG but not absent, as evidenced by a constant increase in ALPL activity during the experimental timeline was registered both in GC and RPM conditions. In accordance with this observation, ELISA results also showed the upregulation at 8 days of SPP1, an osteoblastic marker acting as a mineralization inhibitor [46], and it was demonstrated that its deletion inhibited unloading-related bone loss in mice [47, 48]. Moreover, DAPGs enriching export pathways, and specifically calcium ion export, were downregulated in SMG. Other proteins known to be involved in the mineralization process as components of the matrix vesicles (MVs) are differentially regulated (ANXA6 and NT5DC3 were upregulated, while ATP1A1 and SCARB2 were downregulated) [49]. ATP1A1 was found in vesicles produced by rat osteoblasts, called “osteosomes” [50] and was also found upregulated in human BMSCs differentiation into osteoblasts in vitro, by Granéli et al., together with MME and SLC44A1 [35].

The upregulation at 8 days of SORBS1 and ZYX, enriching for stress fiber assembly, was in line with the results of bioimaging analysis on F-actin. Stress fiber study showed the maximum depolarization (lowest fluorescence intensity) after 1 h of exposure to SMG, as previously shown [51]. The reorganization of the cytoskeleton and reformation of those fibers occurred during the following days (fluorescent area and intensity increased already at day 4 and remained comparable to GC condition). Further upregulation of DAPGs involved in cell migration (ALCAM, NRP2, SEMA3C) suggested BMSCs started migrating again once they reorganized the cytoskeleton and adapted to SMG.

A specific investigation to characterize the energetic and metabolic status of BMSCs during SMG exposure was not performed, although noteworthy PGs were identified within the proteomics data. MT-ATP6, the subunit A of the mitochondrial membrane ATP synthase (F1F0 ATP synthase or Complex V), resulted as downregulated by SMG as previously reported for a different cell type [25], but not for osteoblasts [11]. MT-ATP6 was also found particularly upregulated during the mechanical stimulation of tumoral osteoblasts (MG-63), in connection with an increased oxidative phosphorylation [52]. In our study we showed the downregulation of MT-ATP6 in SMG, as one of the ten mostly downregulated proteins. Furthermore, several other ATP-dependent transporters were downregulated by SMG (ABCC3, ABCC4, ATP1A1, ATP2B1). Guntur et al., recently showed that differentiating immortalized osteoblasts (MT3T3-E1) switch their metabolism on the glycolytic pathway, downregulating oxidative phosphorylation during the differentiation, while 3T3L1 adipocytes stick to the oxidative phosphorylation [53]. In our study, proteins involved in glycolysis, such as PDK1, PDK3 and PKM were found upregulated by SMG, suggesting the energetic metabolism was supporting the osteoblastic differentiation. However, we have also shown that proteins participating in the mitochondrial ß-oxidation (ACOX1, ACOX3, CRAT) were upregulated, suggesting the accumulation of acetyl-CoA and reducing equivalents in SMG, which is more prominent in adipocytes. At the same time, the biosynthesis of both carbohydrates and lipids was downregulated in SMG. The dysregulation of energy metabolism pathways can also be linked to the differential regulation of MTOR and protein glycosylation pathways, an association that has already been investigated in the past for conditions such as obesity, diabetes and cancer [54].

Considering the general picture, we can say that SMG exposure causes an initial acute response consisting in reduced cell adhesion, possibly triggered by a reduction in the pressure perceived by the cells, through the mechanotransduction provided by TMEM63A. This leads to cytoskeletal rearrangements and consequent decline in osteogenic differentiation and osteogenic potential, but it does not cause a complete termination of the osteogenic differentiation. In line with what registered in vivo, under real microgravity [4], our results showed the presence of osteogenic marker expression and mineralization even after 28 days of SMG, in a reduced fashion with respect to the GC condition. Also, the mechanotransduction of SMG stimulus resonates on the energy metabolism, possibly activating it, and therefore preserving the high energy required for osteoblastic differentiation.

It may be possible for us to gain a deeper understanding of the differentiation process by the exploitation of nanomaterials. Our previous studies demonstrated that the application of exogenous calcium hydroxyapatite nanoparticles (nCa-HAPs) control the differentiation kinetics of bone mineralization on BMSCs in physiological conditions. While the application of stable suspensions of strontium hydroxyapatite nanoparticles (nSr-HAPs) [55, 56] would restrict or reverse the molecular changes caused by simulated microgravity in BMSCs exposed to the space environment. Therefore, the functional characterization of nCa- or nSr-HAPs on this in vitro model is required for a detailed molecular knowledge of the system, to understand how comparable the two systems (real vs SMG) might be, and to design new pharmacological countermeasures to treat osteoporosis.

## Conclusions and future perspectives

In this work, the effects of long-term SMG exposure were studied for the first time with a shotgun proteomic approach on a well-characterized in vitro osteoblastic differentiation model based on human primary BMSCs. The influence of SMG on osteoblastic differentiation was highlighted with a temporal pattern analysis: at 8 and 28 days. We can summarize our findings by recounting the two process phases that BMSC undergo in our standardized model when subjected to SMG. There is an initial acute phase, occurring during the first 8 days involving cytoskeleton rearrangement, most probably triggered by the reduced expression of a mechanosensitive calcium channel (TMEM63A or CSC-like protein 1), and a consequent reduction in osteogenic differentiation potential. This is followed by a second phase, from day 8 to day 28, which is characterized by the remarkable plasticity of these cells and which demonstrates that BMSCs in vitro are able to adapt to the stimulus. BMSCs do mineralize the ECM, but their energy metabolism remains altered. We also highlighted how the exposure to SMG was contemporarily affecting a limited number of proteins with respect to the set of proteins regulated by osteogenic factors.

In the future, nanotechnological applications promoting osteogenic differentiation might be used on this model. Furthermore, a new hypothesis involving the coculture of BMSCs with osteocytes, the mechanosensors of the bone, might shed light on the paracrine control of osteoblasts differentiation under SMG. These theories and others need to be the objects of further studies.

## Materials and methods

All chemicals used for this protocol were from Sigma-Aldrich (Germany) if not otherwise specified.

### Isolation of human BMSCs

The study design was approved by the Institutional Ethical Committees of the Fondazione IRCCS Policlinico San Matteo and the University of Pavia (2011). Human BMSCs were isolated from bone marrow (BM) aspirates as previously described [57]. Briefly, BM aspirates were harvested in pediatric clinics from three healthy adult donors, after obtaining written informed consent, and 30 ml of them were designated to BMSCs isolation. Mononuclear cells were isolated from heparinized BM aspirates (30 ml) by Ficoll density gradient (density, 1.077 g/ml; Lymphoprep, Nycomed Pharma, Norway) and plated in non-coated 75–175 cm^2^ polystyrene culture flasks (Corning Costar, Celbio, Italy) at a density of 1.6 ∙ 10^5^ cells/cm. After 48 h, nonadherent cells were discarded and culture medium was replaced twice a week. Once the 80% of confluence was reached, the cells were harvested and replated for expansion at a density of 4000 cells/cm^2^ in a continuous way until the third passage. Up to this point, cells were cultured in mesencult medium (Stem Cell Technologies, Canada) supplemented with 2-mM L-glutamine, 50-mg/ml gentamicin, and 10% v/v FCS. Cultures were maintained at 37 °C in a humidified and CO_2_ (5%) conditioned atmosphere. According to the International Society for Cellular Therapy on the nomenclature of mesenchymal progenitors, the cells cultured for this study were defined as multipotent stromal cells. To phenotypically characterize BMSCs and to define their purity, FACS analysis was performed as previously indicated [57]. Following this point, osteogenic differentiation began, as explained in the next section.

### Osteogenic differentiation of BMSCs in SMG

Following expansion, BMSCs were plated on different supports (4 well plate, 8-well tissue culture treated µ-Slides (Ibidi, Germany) or 25 cm^2^ polystyrene culture flasks), according to the type of assay. Seeding was performed at a cell density of 3.5 × 10^4^ cells/cm^2^, in complete mesencult medium and cells were cultured for 3 days in a humidified atmosphere, at 37 °C, with 5% CO_2_. T0 was set on the third day of culture when all cell supports were fully filled with osteogenic medium (OM), sealed with sterile parafilm and air bubbles removed with syringes. The osteogenic medium was composed by α-MEM (Minimum Essential Medium) (ECM0849L, Euroclone) supplemented with 10% FBS (FBS,180 l, Lonza), 2-mM L-glutamine, 2% v/v sodium pyruvate, 1% v/v penicillin–streptomycin, 0.2% v/v fungizone, 2% HEPES, and three osteogenic factors: 100-nM dexamethasone, 5-mM β-glycerophosphate, and 50-mg/ml ascorbic acid. The medium was changed every 4 days; to allow medium renewal of the SMG samples, the RPM was stopped for less than 5 min every time. The differentiation was definitely terminated at T28. Osteogenic differentiation was studied directly and contemporary in GC condition and in SMG, by using the RPM (Dutch Space) [14, 55]. Half of the cell vessels were exposed to SMG on the RPM and half were positioned on the static support of RPM as GC, until T28. The RPM conditions were settled as previously described [55].

### Cell viability assay

Cell viability was monitored at the two most relevant time points for the osteogenic differentiation: T8 and T28, both in GC and RPM. Earlier time points were evaluated in previous experiments, and showed no differences between SMG and GC [55]. T8 and T28 were also used for all the subsequent assays. Trypan blue was used to count the number of alive cells in each condition. In the chart (Fig. S1), the percentage of living cells is plotted with respect to the total number of seeded cells.

### ALPL activity assay

ALPL activity assay was performed at 8, 14 (T14), 21 (T21) and 28 days on differentiating BMSCs, both in GC and RPM. Three wells per each condition were incubated for 30 min at 37 °C with 0.3 M substrate *p*-nitrophenyl phosphate (*p-*NPP) (Sigma-Aldrich, Germany) dissolved in a glycine buffer (pH 10.5). Titration curve was incubated for 10 min with 14 U/ml of recombinant ALPL (Sigma-Aldrich, Germany) and 0–50 μM of *p*-NPP. Active ALPL was able to transform *p*-NPP in *p*-nitrophenyl (*p-*NP) upon reaction stop by 5 M NaOH. Solution absorbance was measured at 450 nm in a colorimetric 96-well plate, with TECAN Infinite^®^ F500 microplate reader (TECAN, Switzerland). Samples and standards were read in triplicate, and the standard curve was used for unknown sample concentration calculation.

### Quantitative real-time PCR

To compare gene expression during differentiation, the same number of cells was cultured in GC and RPM. Cells were harvested at T8 and T28 and total RNA was extracted with the RNeasy Plus Mini Kit (Qiagen, Hilden, Germany). Reverse transcription was performed on 500 ng of total RNA by high-capacity cDNA Reverse Transcription Kit (Thermo Fisher Scientific, Carlsbad, US). Quantitative real time PCR (qRT-PCR) was performed in a 96-well optical reaction plate in a 7500 Fast Real-Time PCR System (Applied Biosystems, Germany) using single tube Taqman real-time PCR assays (Thermo Fisher Scientific, Carlsbad, US). Each assay included triplicate reactions. A negative control without template was run with each assay to assess the overall specificity. The relative abundance of each gene was determined by the 2^−ΔΔCt^ method [58], and data were presented as a fold change in gene expression normalized to the endogenous control gene, the *18S* rRNA, and relative to GCs. Quantitative results are expressed as the mean ± standard deviation. Student’s T test was applied with a significance level of 0.05 to compare results.

### Crystal size study

Images for the morphological analysis of hydroxyapatite crystals were collected on live cell samples. Images were acquired over 28 days (T1, T8, T14, T21, T28) BMSCs osteogenic differentiation by a Nikon Eclipse Te200 (Nikon, Japan) equipped with 20 × magnification and a Nikon Ds-Fi1 camera (sense: 5 MP CMOS) controlled by a Ds-U3camera. Pre-calibrated images were analyzed with ImageJ and single deposited hydroxyapatite crystals were manually segmented in order to obtain crystal area. Statistical analysis was performed comparing areas of crystals grown in GC and RPM with a Student’s *T* test (*p* < 0.05), and frequency distribution of the analyzed crystals was also calculated.

### Alizarin Red assay

ECM calcium deposition was measured with Alizarin Red assay at T8 and T28, in GC and RPM conditions [55]. Samples cultured in four well plates were fixed with 4% paraformaldehyde (PFA) for 30 min at 4 °C and stained with 40 mM of Alizarin Red (pH 4.2) for 1 h at RT to analyze the calcium deposits. Samples were rinsed 3 times in distilled water for 10 min each. Images were acquired by Nikon Eclipse Te2000 equipped with 10 × or 20 × magnification and a Nikon Ds-Fi1 camera. Alizarin Red staining was quantified after incubation with 10% cetylpyridinium chloride (Sigma-Aldrich, Germany) in 10 mM sodium phosphate (pH 7.0) for 20 min at RT. Solution absorbance was measured in triplicate with TECAN Infinite^®^ F500 microplate reader at 562 nm.

### Extracellular matrix protein composition assessment by ELISA

At T8 and T28 the concentration of ECM proteins was evaluated in samples grown in GC and RPM by ELISA. Samples were washed with sterile phosphate buffer saline (PBS) and incubated with sterile lysis buffer (20 mM Tris–HCl, 4 M GuHCl, 10 mM EDTA, pH 8.0) for 24 h at 37 °C. The total protein concentration of the different samples was evaluated via Bicinchoninic acid (BCA) Protein Assay Kit (Pierce Biotechnology, Rockford, IL, USA). Primary antibodies used for the ELISA: Dr. Larry W. Fisher (Fisher) provided us with the primary rabbit polyclonal antibodies against COL1A1 (LF-68), COL3A1 (LF-70), DCN (LF-136), BGLAP (LF-126), SPARC (LF-37), and SPP1 (LF-86). Antibody stock solutions were prepared as suggested by the literature. Rabbit polyclonal antibody against human FN was produced as previously described [59]. For the calibration curves, microtiter wells were coated with increasing concentrations (10 ng to 2 μg) of purified protein, in a coating buffer (50 mM Na_2_CO_3_, pH 9.5) overnight at 4 °C. For sample assessment, microtiter wells were coated with previously extracted ECM proteins (20 μg/mL in coating buffer) overnight at 4 °C. Control microtiters wells were coated with BSA. After three washes with PBS containing 0.1% (v/v) Tween 20, the wells were blocked by incubating them with 2% (w/v) BSA in PBS for 2 h at 22 °C. The wells were subsequently incubated with primary antibodies (1:500 dilution in 1% BSA) for 1.5 h at 22 °C. After washing, the wells were incubated for 1 h at 22 °C with goat anti-rabbit IgG (1:1000 dilution in 1% BSA) conjugated to horseradish peroxidase (HRP). Reaction development was achieved with o-phenylenediamine dihydrochloride substrate in phosphate-citrate buffer. The reaction was stopped with 0.5 M H_2_SO_4_, and the absorbance was measured at 490 nm with a microplate reader (BioRad Laboratories, Germany).

### Extracellular matrix protein immunostaining and bioimaging analysis

BMSCs osteogenic differentiation cultures were stained for ECM proteins collagen-type I and BGLAP using indirect immunofluorescence, whereas nuclei were revealed by direct fluorescence. At the T8 and T28 samples in eight well µ-Slide differentiated on RPM and in GC were fixed with 4% PFA at 4 °C for 15 min. Samples were blocked with PAT (PBS containing 1% [w/v] bovine serum albumin and 0.02% [v/v] Tween 20) for 120 min at RT. Fluorescent staining was performed by exposing the samples to rabbit polyclonal antibodies against COL1A1 (LF-68, 1:250) or BGLAP (LF-126, 1:250) for 2 h at 37 °C. Following incubation with fluorescein isothiocyanate (FITC)-conjugated anti-rabbit IgG for 120 min at 37 °C in the dark, permeabilization with 0.1% Triton X-100 and counterstain with DAPI (4’,6-Diamidino-2-Phenylindole, Biochemica, STATE) (1:1000) were performed. Samples were finally rinsed twice with distilled water and fluorescence images were acquired by Zeiss Axio Observer with 20X coupled with camera QImaging Retiga (1.5 MP monochromatic CCD sensor) and Image-pro Plus software. Image analysis of COL1A1 and BGLAP were performed with ImageJ by measuring total intensity and area per each image. Student’s *T* test was used in all experiments and the significance level was set to *p* < 0.05 for all statistical analyses.

### Cytoskeleton immunostaining and bioimaging analysis

F-Actin and β-Tubulin staining was performed at T1h, T1, T4, T8, T14, and T28. At each time point, samples in eight well µ-Slides were fixed with PFA 4% at 4 °C for 15 min and stored in PBS at 4 °C until staining. Samples were permeabilized with 0.1% Triton X-100 for 3 min at RT and blocked with PAT (PBS containing 1% [w/v] bovine serum albumin and 0.02% [v/v] Tween 20) for 120 min at RT. Indirect fluorescent staining was performed by exposing samples to primary mouse monoclonal antibody against β-Tubulin (Sigma-Aldrich) (1:100) for 1 h at RT followed by 3 rinse in PBS and secondary FITC-conjugated anti-mouse IgG (1:100) for 1 h at 37 °C in dark conditions. Direct staining was performed by exposing samples to TRITC-conjugated Phalloidin (Sigma-Aldrich) (1:1000) for 15 min. Finally, counterstaining with DAPI (Biochemica, STATE) (1:1000) was performed. After slides mounting, samples fluorescence images were acquired by Zeiss Axio Observer with 40X coupled with camera QImaging Retiga (1.5 MP monochromatic CCD sensor) and Image-pro Plus software.

Image analysis was performed with ImageJ by measuring mean intensity and fraction area into cells of F-Actin and β-Tubulin. Additionally, β-Tubulin distribution from the centrosome to the cell periphery was initially evaluated by “fire lookup table” and later by a developed algorithm which generated concentric and equally distributed belts from the center to the cell edge where the cell edge was manually drawn and the centrosome was assigned by the highest intensity point of microtubule network into cell. In each belt, mean intensity and area fraction of β-Tubulin were determined. Statistical analyses were performed with Student’s T test was applied with a significance level of 0.05.

### Proteome extraction and in-gel digestion

At T0, T8, and T28 of differentiation cells were lysed using a 0.1% deoxycholate, 0.1% *n*-octyl-β-d-glucoside, 0.1 M TRIS–HCl (pH 7.5), 1 mM EDTA and 0.1 M NaCl buffer. Three biological replicates were made for each condition, except for T8 GC in which only two were lysed and further processed, making a total of 14 samples. Benzonase nuclease was added to each sample to dissolve DNA clumps during a 30 min incubation at 37 °C. Proteins were precipitated in acetone and stored at − 80 °C until further processing. Protein resuspension was performed in 2X Laemmli buffer (125 mM Tris–HCl pH 6.8, 2% SDS, 16% glycerol, 0.16 M dithiothreitol, 0.01% bromophenol blue) and protein concentration of all samples was assessed by Pierce 660 nm protein assay reagent with the addition of the ionic detergent compatibility reagent (Thermo Scientific, Germany) to prevent SDS interference. A total of 10 μg of proteins per sample was run on 12% handcrafted gels, and SDS-PAGE was performed to concentrate each sample in a single band. Gel bands were excised, reduced with 20 mM dithiothreitol at 56 °C, alkylated with 55 mM iodoacetamide at RT in the dark and digested with mass spectrometry grade trypsin (Promega, Germany). Trypsin was added at a ratio of 1:30 with respect to the total protein content of each band. Following digestion at 37 °C for 16 h, trypsin was inactivated by addition of formic acid at a final concentration of 0.2%. Peptides were eluted twice from gel pieces, using 100% acetonitrile and 50% acetonitrile/5% formic acid. Eluted aliquots were vacuum dried in SpeedVac (Thermo Fisher Scientific, Germany). Dried peptides were resuspended in 0.1% formic acid and peptide yield was checked with Pierce™ Quantitative Fluorometric Peptide Assay (Thermo Fisher Scientific, Germany).

### Label-free-quantification by Evosep one liquid chromatography—tandem mass spectrometry analysis and data-independent acquisition

For each sample, 800 ng of peptides were loaded on Evotip disposable trap columns (Evosep, Denmark) according to manufacturer’s instructions to perform label-free quantification via nano-liquid chromatography-tandem mass spectrometry (LFQ nLC-MS/MS). Peptides were separated on a 44 min gradient in Evosep One UHPLC system (Evosep, Denmark)—reversed phase chromatography—connected to Orbitrap Fusion Lumos Mass Spectrometer (Thermo Scientific). Xcalibur software (Thermo Scientific, Germany) was used for setting the instrument parameters. The first mass spectrometry (MS1) scans were obtained at 120,000 *m*/*z* resolution in Orbitrap, in 390–810 *m*/*z* scan range, with 500,000 AGC target and 20 ms maximum injection time. Data-independent acquisition (DIA) scans were performed in Orbitrap at 30 K resolution with 1,000,000 AGC target, 60 ms maximum ion injection time and fragmentation was performed in HCD mode with 27% collision energy. Thirty-two variable width data-independent acquisition (DIA) windows were adjusted for equal precursor ion density in the 399–800 *m*/*z* scan range.

### Protein groups identification

Raw data from the 32 windows scanning the 399–800 *m*/*z* range were analyzed on Spectronaut 15 (Biognosys, Switzerland) in a single batch in direct DIA mode. Homosapiens uniprotKB database with isoforms (downloaded 09.09.2020) was used for generating the search database, and Trypsin/P with maximum two miss-cleavages allowed was used for digesting the proteins. Carbamidomethylation of Cys residues was set as fixed modification and acetylation (protein-term), oxidation of Met and deamidation of Asn-Gln were set as variable modifications. Data were quantified in Spectronaut with default settings and median normalized. The list of 4312 identified protein groups was used for the subsequent statistical analysis (Table S1). The mass spectrometry proteomics data have been deposited to the ProteomeXchange Consortium via the Proteomics Identifications Database—EMBL-EBI (PRIDE) [60] partner repository with the dataset identifier PXD033475.

### Statistical preprocessing, processing, and enrichment of proteomics data

Statistical preprocessing and processing were performed with Perseus software (1.6.13.0) [61, 62] and Orange Data Mining software (3.27.1) [63] was used for data mining and visualization. Data were initially categorized based on the time of collection (T0, T8 and T28) and the culturing conditions (GC and RPM) of the samples, resulting in five different groups (each with three replicates, except for the T8_GC which only had two). The categorized data went through several steps: 1. the intensities of each protein group (PG) in the T0 replicates were averaged; 2. PGs intensities of all samples (except the T0) were rationed on the T0 mean intensities and T0 replicates were not considered for subsequent analysis; 3. The matrix was filtered for valid values (70% in at least one category), log2 transformed and missing data were imputed on the base of their Gaussian distribution (Fig. S2). One-way ANOVA was applied at this point to retrieve statistically significant differences among PG intensities relative to the four groups (*p* < 0.05). Furthermore, the correction for multiple-dependent variables is considered by the Perseus software, and we used Permutation-based False Discovery Rate, with 250 repetitions, accepting a 5% of false-positive DAPGs (error rate = 0.05) [62]. Multiple comparisons (Tukey’s honestly significant difference test; *p* < 0.05) was run on the 486 differentially abundant PGs (DAPGs) and average differences were annotated. The PGs and DAPGs were labeled using the official gene names, which were univocally recognizable by the different software and repositories used. The 486 DAPGs were manually controlled to remove redundant gene names referring to uncharacterized isoforms. The list was, therefore, reduced to 481 DAPGs (Table S2). GO annotation overrepresentation analysis (ORA) of the 481 DAPGs for the cellular component categories was performed with the WebGestalt online tool, setting a significant threshold at *p* value < 0.05. Following gene ontology (GO) term annotation, the DAPGs were divided in four lists: up- and down-regulated by SMG, either at 8 or 28 days of differentiation. Functional enrichment analysis of the GO biological processes (GO_BPs) of each list was performed with GeneSCF [64] (Fig. S4, S5, S6, S7). GO_BPs were then grouped in macro-categories of biological process: cell fate (comprising proliferation, differentiation, adhesion, signaling, and death) and cell metabolism (including carbohydrates, lipids, proteins, transport, and nucleic acids) (Fig. 5). A principal component analysis (PCA) was performed (Fig. 4B) on the variance stabilizing transformed matrix using DESeq2 v1.30.1 [65].

## Supplementary Information

Below is the link to the electronic supplementary material.

Supplementary file1 Fig S1 BMSCs viability in GC and RPM. The number of alive cells was indirectly determined with trypan blue exclusion. Data have been related to the number of seeded cells at the beginning of the experiment. (TIFF 1982 KB)

Supplementary file2 Fig S2 Statistical samples distribution. Frequency distribution of the protein intensities shaping gaussian curves for each of the analyzed sample. (TIFF 618 KB)

Supplementary file3 Fig S3 Enrichment of the GO-Cellular Components. On the X-axis the names of the GO-CC terms that were significantly enriched. On the Y-axis the enrichment ratios have been plotted and the numeric p-values relative to each category are specified on top of each bar. (TIFF 6228 KB)

Supplementary file4 Fig S4 Enrichment of the GO biological process based on the DAPGs downregulated at 8 days of differentiation. On the horizontal axis the percentage of enrichment has been reported. Blue bars represent the 66 pathways that were significantly downregulated in RPM. (TIFF 31281 KB)

Supplementary file5 Fig S5 Enrichment of the GO biological process based on the DAPGs upregulated at 8 days of differentiation. On the horizontal axis the percentage of enrichment has been reported. Red bars represent the 28 pathways that were significantly downregulated in RPM. (TIFF 25163 KB)

Supplementary file6 Fig S6 Enrichment of the GO biological process based on the DAPGs downregulated at 28 days of differentiation. On the horizontal axis the percentage of enrichment has been reported. Blue bars represent the 40 pathways that were significantly downregulated in RPM. (TIFF 31467 KB)

Supplementary file7 Fig S7 Enrichment of the GO biological process based on the DAPGs upregulated at 28 days of differentiation. On the horizontal axis the percentage of enrichment has been reported. Red bars represent the 62 pathways that were significantly downregulated in RPM. (TIFF 26368 KB)

Supplementary file8 Table S1 Samples report from Spectronaut software. List of the identified and normalized protein groups per each sample. Samples names are composed by the time point (“Tn”, where n is the day of differentiation), the condition in which they were kept (GC= Gravity Control; RPM= simulated microgravity), and the number of the replicate. Column name specification: “N: [sample_number] 201218_DF_nn_1.raw.PG.NrOfPrecursorsIdentified – sample name” = number of precursor ions used for the identification of each protein group; “T: PG.Pvalue” = the p-value of the Protein Group identification; “T: PG.ProteinGroups” = Uniprot accession numbers of the identified protein groups; “T: PG.Genes” = gene symbols of the identified protein groups. (XLSX 1059 KB)

Supplementary file9 Table S2 List of the positive hits from the One-way ANOVA with relative multiple comparison results. Column name specification: “C: ANOVA Significant” = with “+” we indicated a positive significance; “N: -Log ANOVA p value” = the –log(2) of the one-way ANOVA p-value; “T: PG.Names” = extended name of the protein group; “T: Significant pairs” = significant comparisons calculated with Tukey’s Honestly Significant Differences (p<0.05). (XLSX 136 KB)

Supplementary file10 Table S3 DAPGs (ANOVA positive hits) were divided in four groups, specified in the first column: T8_up/T28_up = upregulated by SMG at T8/T28; T8_down/T28_down = downregulated by SMG at T8/T28. For each group a separate enrichment was performed using GeneSCF (XLSX 61 KB)

## Data Availability

The proteomics data have been deposited in the PRIDE database under the identification number PXD033475 and under the project name: “Human bone marrow stromal cells under simulated microgravity’ bottom-up, label free, shotgun approach in DIA”. All other data that support the findings of this study are available from the corresponding author upon reasonable request.
