# Supplementary figures and images for: Long-term osteogenic differentiation of human bone marrow stromal cells in simulated microgravity: novel proteins sighted
Source: Cell Mol Life Sci. 2022 Oct 1;79(10):536. doi: 10.1007/s00018-022-04553-2 (PMC9526692; doi:10.1007/s00018-022-04553-2)

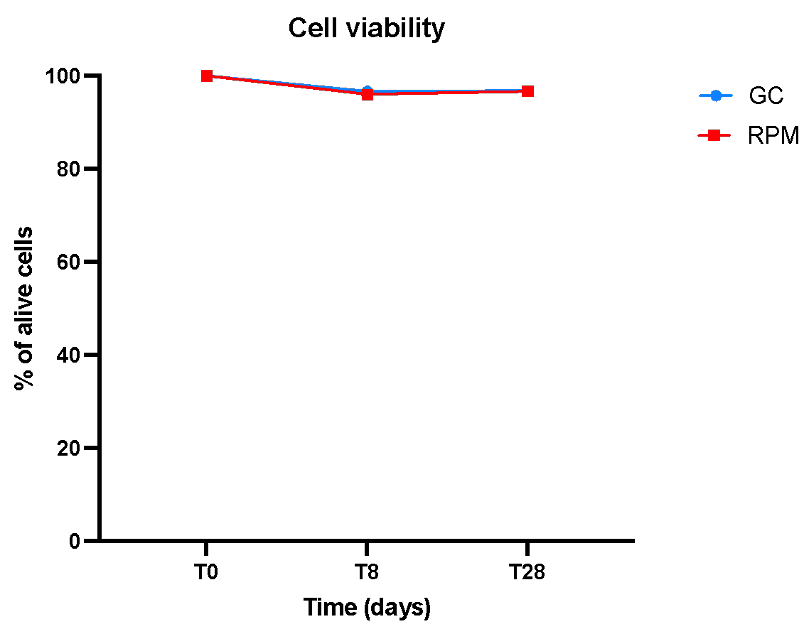

Supplement: Supplementary file 1 — Supplementary file1 Fig S1 BMSCs viability in GC and RPM. The number of alive cells was indirectly determined with trypan blue exclusion. Data have been related to the number of seeded cells at the beginning of the experiment. (TIFF 1982 KB) [file 18_2022_4553_MOESM1_ESM.tiff]

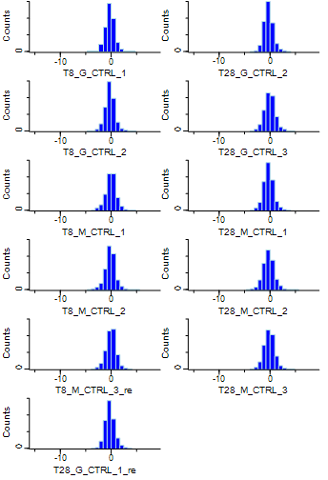

Supplement: Supplementary file 2 — Supplementary file2 Fig S2 Statistical samples distribution. Frequency distribution of the protein intensities shaping gaussian curves for each of the analyzed sample. (TIFF 618 KB) [file 18_2022_4553_MOESM2_ESM.tiff]

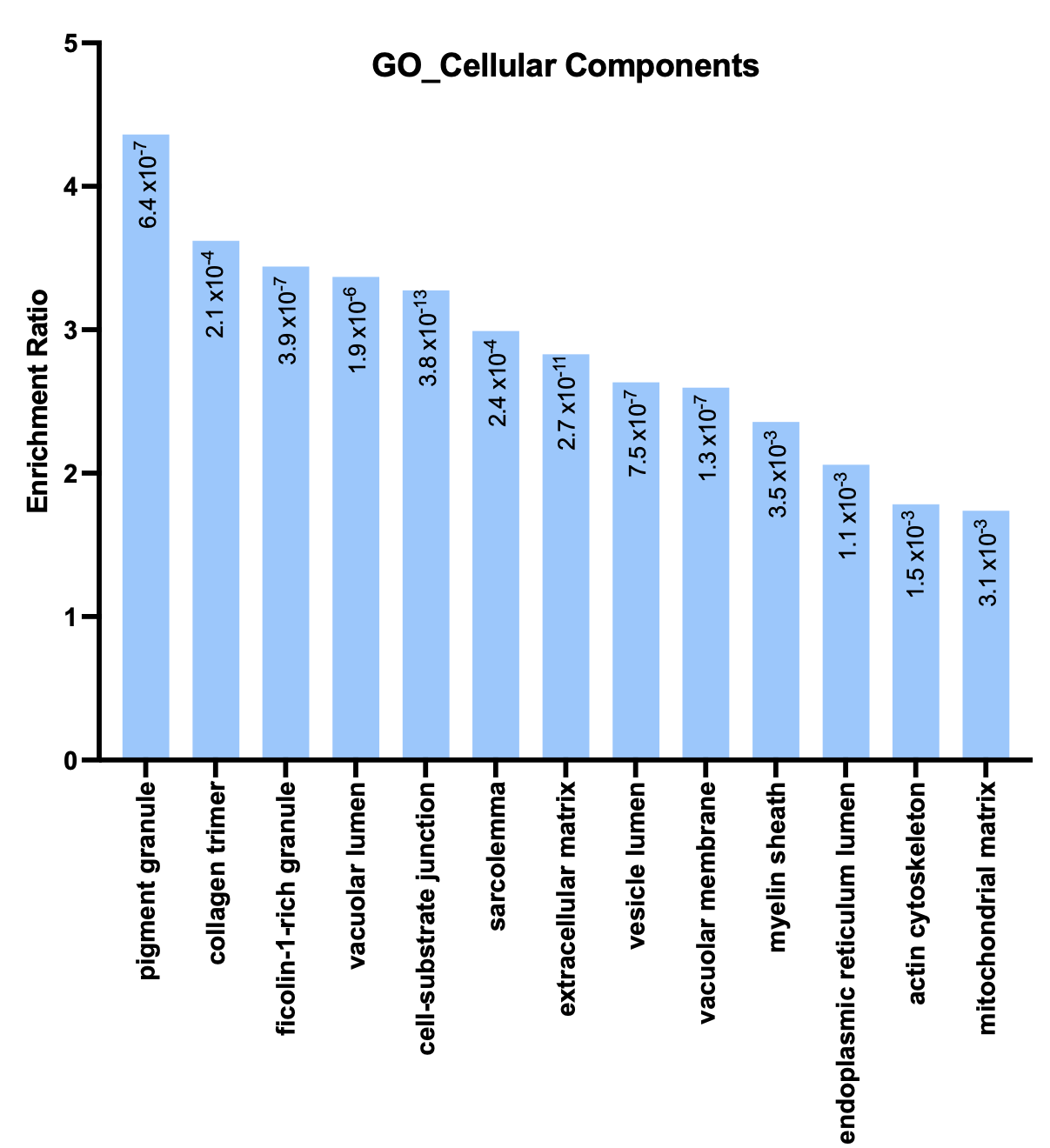

Supplement: Supplementary file 3 — Supplementary file3 Fig S3 Enrichment of the GO-Cellular Components. On the X-axis the names of the GO-CC terms that were significantly enriched. On the Y-axis the enrichment ratios have been plotted and the numeric p-values relative to each category are specified on top of each bar. (TIFF 6228 KB) [file 18_2022_4553_MOESM3_ESM.tiff]

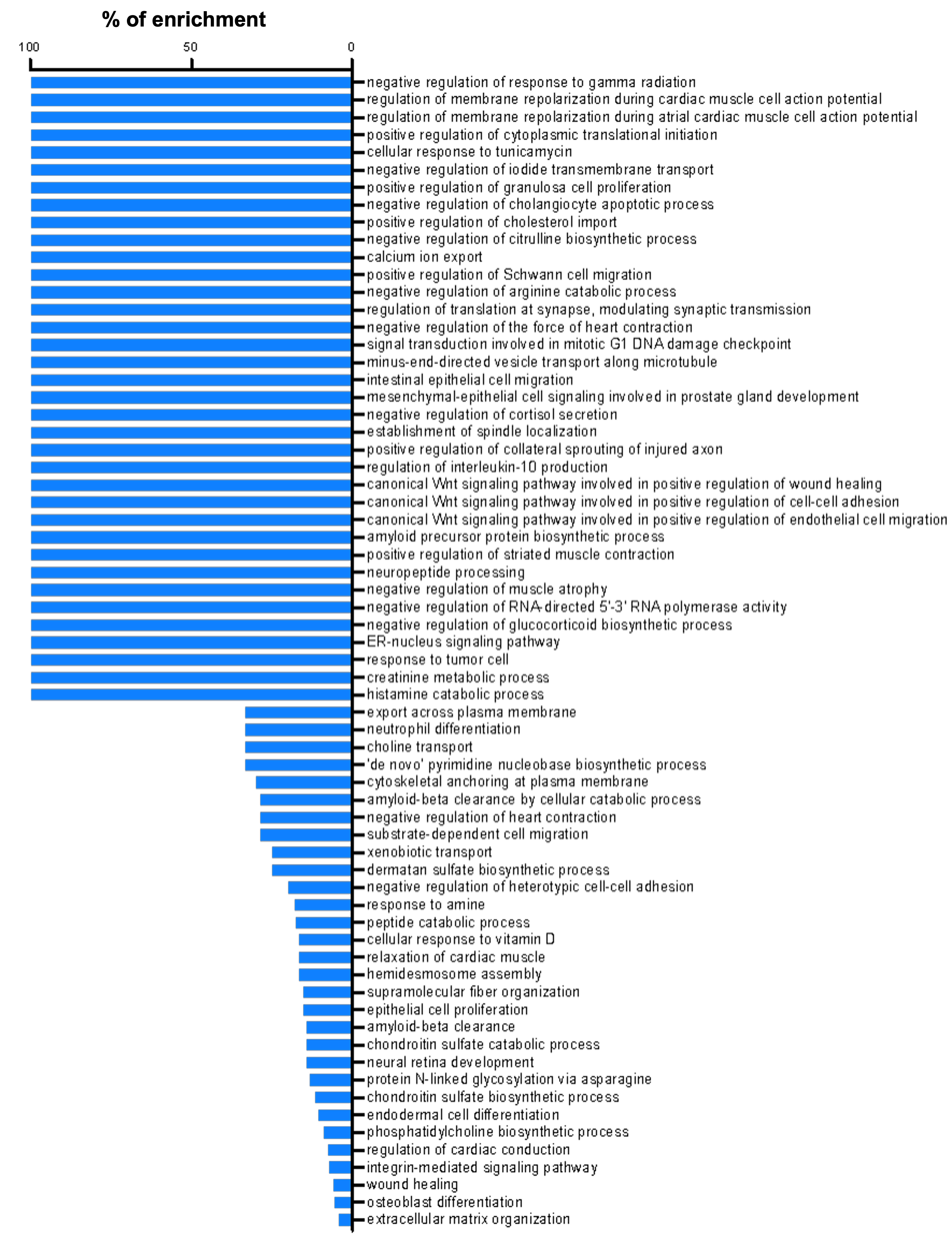

Supplement: Supplementary file 4 — Supplementary file4 Fig S4 Enrichment of the GO biological process based on the DAPGs downregulated at 8 days of differentiation. On the horizontal axis the percentage of enrichment has been reported. Blue bars represent the 66 pathways that were significantly downregulated in RPM. (TIFF 31281 KB) [file 18_2022_4553_MOESM4_ESM.tiff]

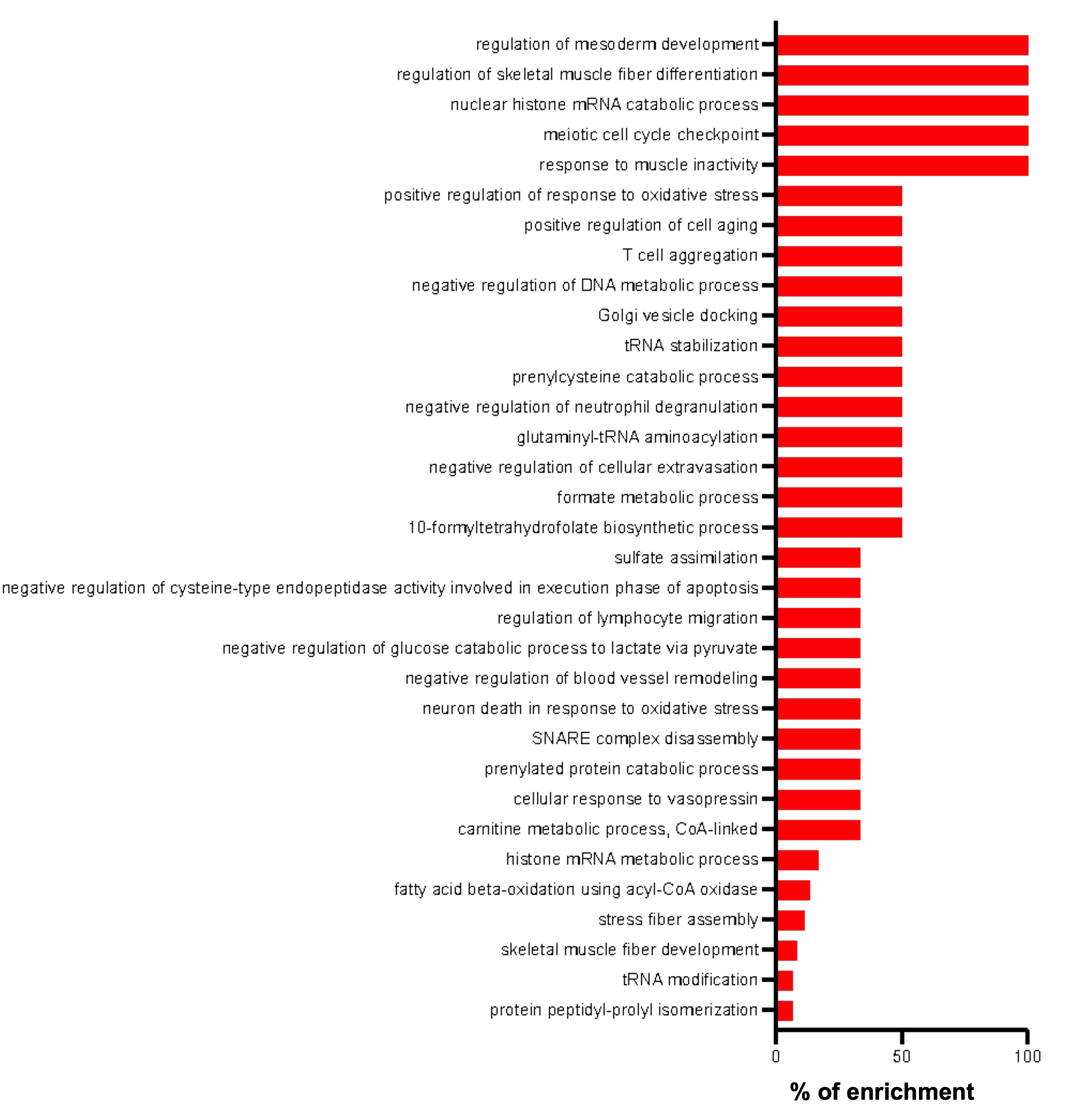

Supplement: Supplementary file 5 — Supplementary file5 Fig S5 Enrichment of the GO biological process based on the DAPGs upregulated at 8 days of differentiation. On the horizontal axis the percentage of enrichment has been reported. Red bars represent the 28 pathways that were significantly downregulated in RPM. (TIFF 25163 KB) [file 18_2022_4553_MOESM5_ESM.tiff]

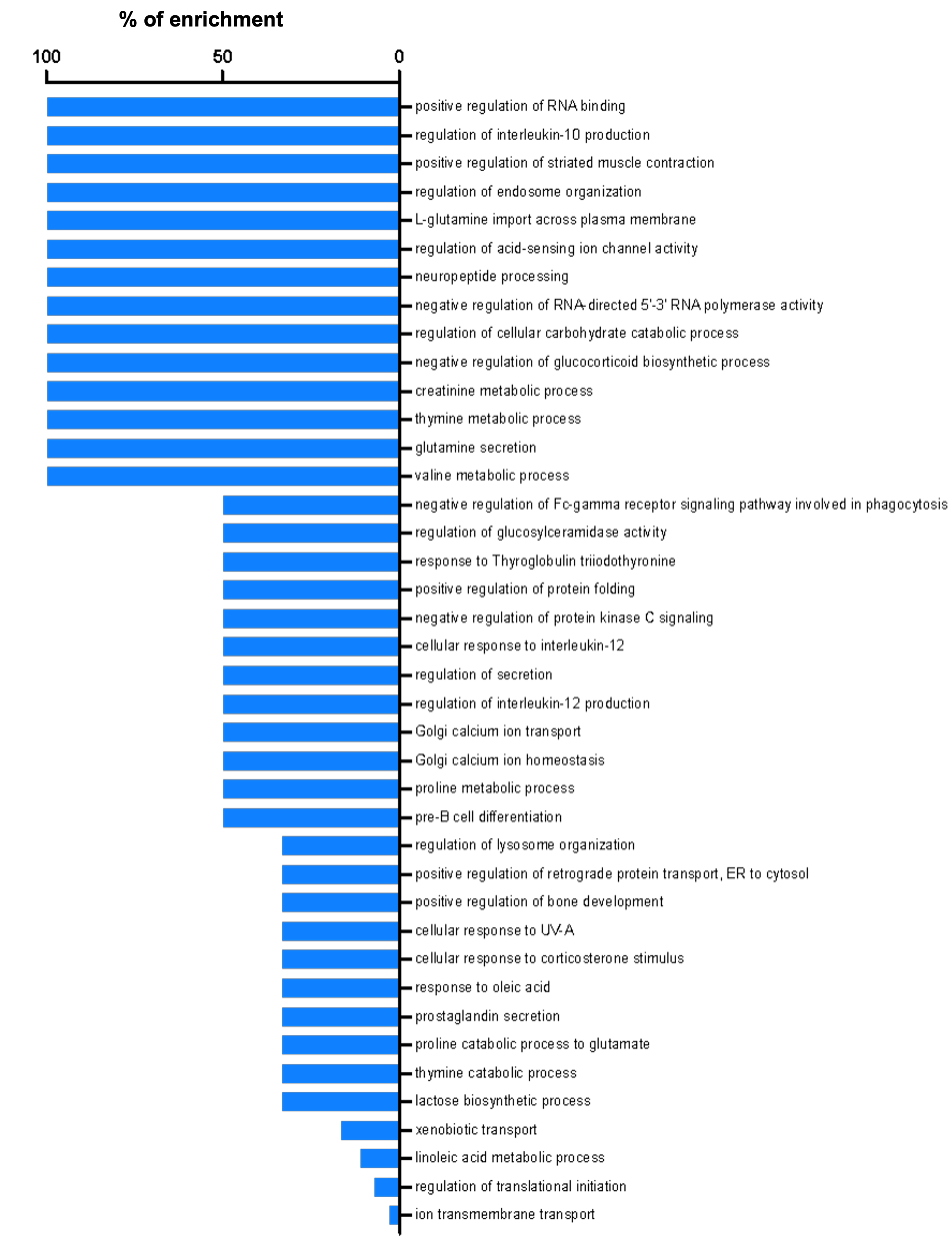

Supplement: Supplementary file 6 — Supplementary file6 Fig S6 Enrichment of the GO biological process based on the DAPGs downregulated at 28 days of differentiation. On the horizontal axis the percentage of enrichment has been reported. Blue bars represent the 40 pathways that were significantly downregulated in RPM. (TIFF 31467 KB) [file 18_2022_4553_MOESM6_ESM.tiff]

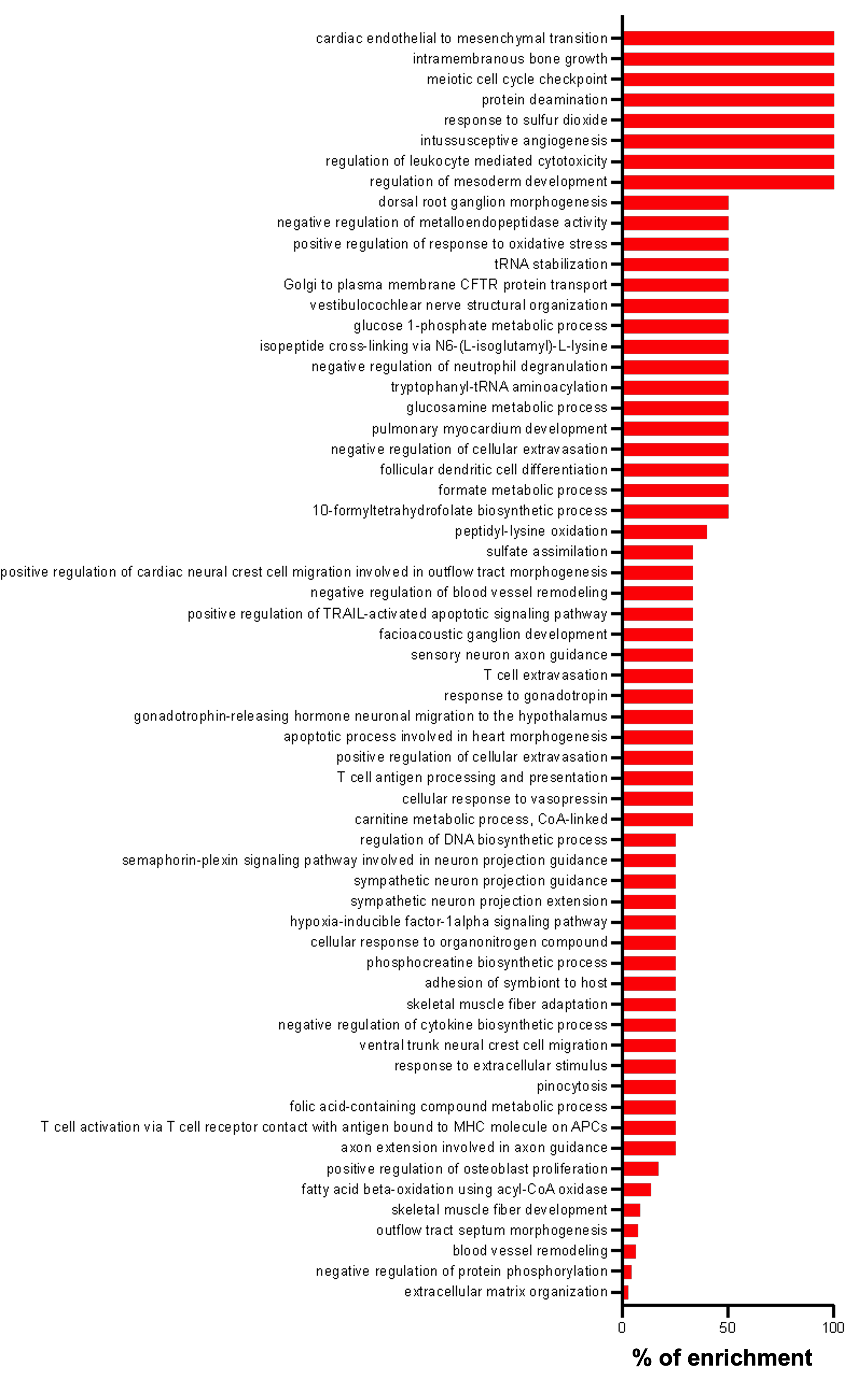

Supplement: Supplementary file 7 — Supplementary file7 Fig S7 Enrichment of the GO biological process based on the DAPGs upregulated at 28 days of differentiation. On the horizontal axis the percentage of enrichment has been reported. Red bars represent the 62 pathways that were significantly downregulated in RPM. (TIFF 26368 KB) [file 18_2022_4553_MOESM7_ESM.tiff]
